# Supplementary material for: Deposition of Nanosized Amino Acid Functionalized Bismuth Oxido Clusters on Gold Surfaces
Source: Nanomaterials (Basel). 2022 May 26;12(11):1815. doi: 10.3390/nano12111815 (PMC9182479; doi:10.3390/nano12111815)
Supplement: Supplementary file 1 [file nanomaterials-12-01815-s001.zip › nanomaterials-1692046-supplementary.pdf]

nanomaterials

Supplementary Materials

Deposition of Nanosized Amino Acid Functionalized Bismuth Oxido Clusters on Gold Surfaces

Annika Morgenstern [1], Rico Thomas [2], Apoorva Sharma [1], Marcus Weber [2,3], Oleksandr Selyshchev [1], Ilya Milekhin [1], Doreen Dentel [4], Sibylle Gemming [3,5], Christoph Tegenkamp [4], Dietrich R. T. Zahn [1,3], Michael Mehring [2,3,*] and Georgeta Salvan [1,3,*]

[1] Semiconductor Physics, Institute of Physics, Chemnitz University of Technology, 09107 Chemnitz, Germany;
annika.morgenstern@physik.tu-chemnitz.de (A.M.); apoorva.sharma@physik.tu-chemnitz.de (A.S.); oleksandr.selyshchev@physik.tu-chemnitz.de (O.S.); ilya.milekhin@physik.tu-chemnitz.de (I.M.); zahn@physik.tu-chemnitz.de (D.R.T.Z.)
[2] Coordination Chemistry, Institute of Chemistry, Chemnitz University of Technology, 09107 Chemnitz, Germany; rico.thomas@s2013.tu-chemnitz.de (R.T.); marcus.weber@chemie.tu-chemnitz.de (M.W.)
[3] Center of Materials, Architectures and Integration of Nanomembranes, Chemnitz University of Technology, 09126 Chemnitz, Germany, sibylle.gemming@physik.tu-chemnitz.de
[4] Solid Surface Analysis, Institute of Physics, Chemnitz University of Technology, 09107 Chemnitz, Germany; doreen.dentel@physik.tu-chemnitz.de (D.D.); christoph.tegenkamp@physik.tu-chemnitz.de (C.T.)
[5] Theoretical Physics of Quantum Mechanical Processes and Systems, Institute of Physics, Chemnitz University of Technology, 09107 Chemnitz, Germany
* Correspondence: michael.mehring@chemie.tu-chemnitz.de (M.M.); salvan@physik.tu-chemnitz.de (G.S.)

Citation: Morgenstern, A.; Thomas, R.; Sharma, A.; Weber, M.; Selyshchev, O.; Milekhin, I.; Dentel, D.; Gemming, S.; Tegenkamp, C.; Zahn, D.R.T.; et al. Deposition of Nanosized Amino Acid Functionalized Bismuth Oxido Clusters on Gold Surfaces. *Nanomaterials* **2022**, *12*, 1815. https://doi.org/10.3390/nano12111815

Academic Editor(s): Valentin Craciun, Felicia Iacomi and Romulus Tetean

Received: 5 April 2022
Accepted: 19 May 2022
Published: 26 May 2022

Publisher's Note: MDPI stays neutral with regard to jurisdictional claims in published maps and institutional affiliations.

Copyright: © 2022 by the authors. Licensee MDPI, Basel, Switzerland. This article is an open access article distributed under the terms and conditions of the Creative Commons Attribution (CC BY) license (https://creativecommons.org/licenses/by/4.0/).

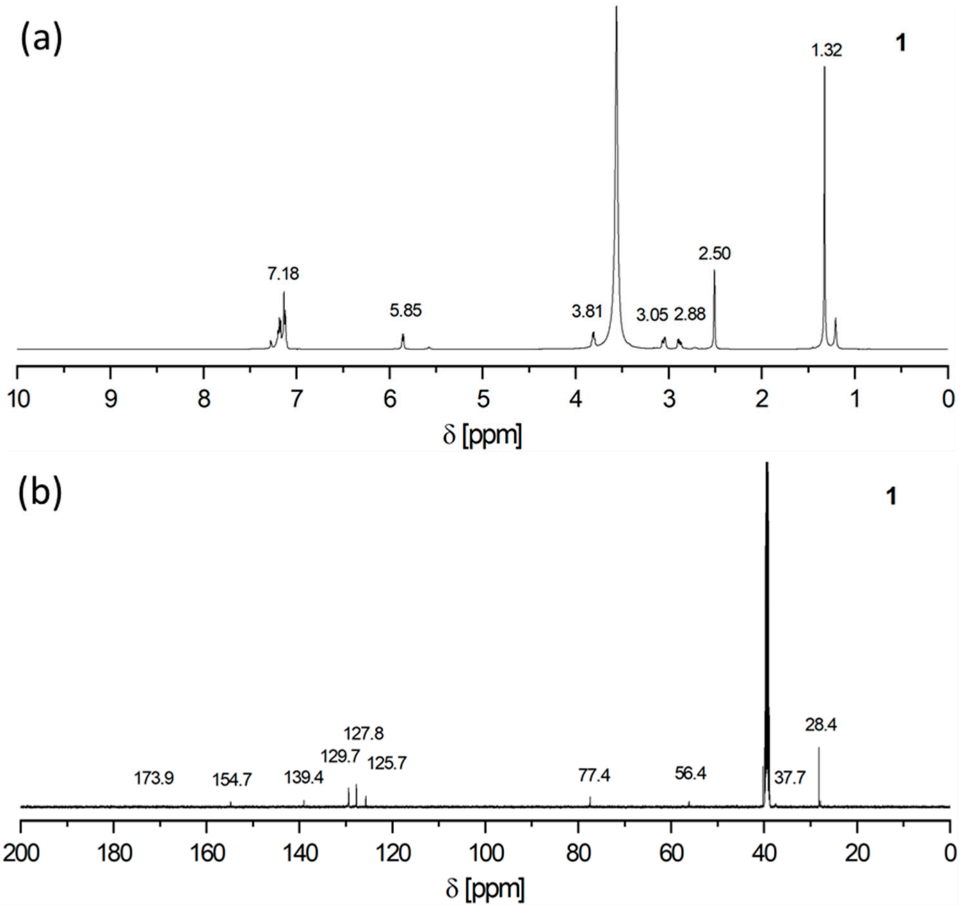

Figure S1. (**a**) ^{1}H NMR spectra (500.3 MHz, 298 K) of **1** in dmso-d$_6$. (b) ^{13}C NMR spectra (125.8 MHz, 298 K) of **1** in dmso-d$_6$.

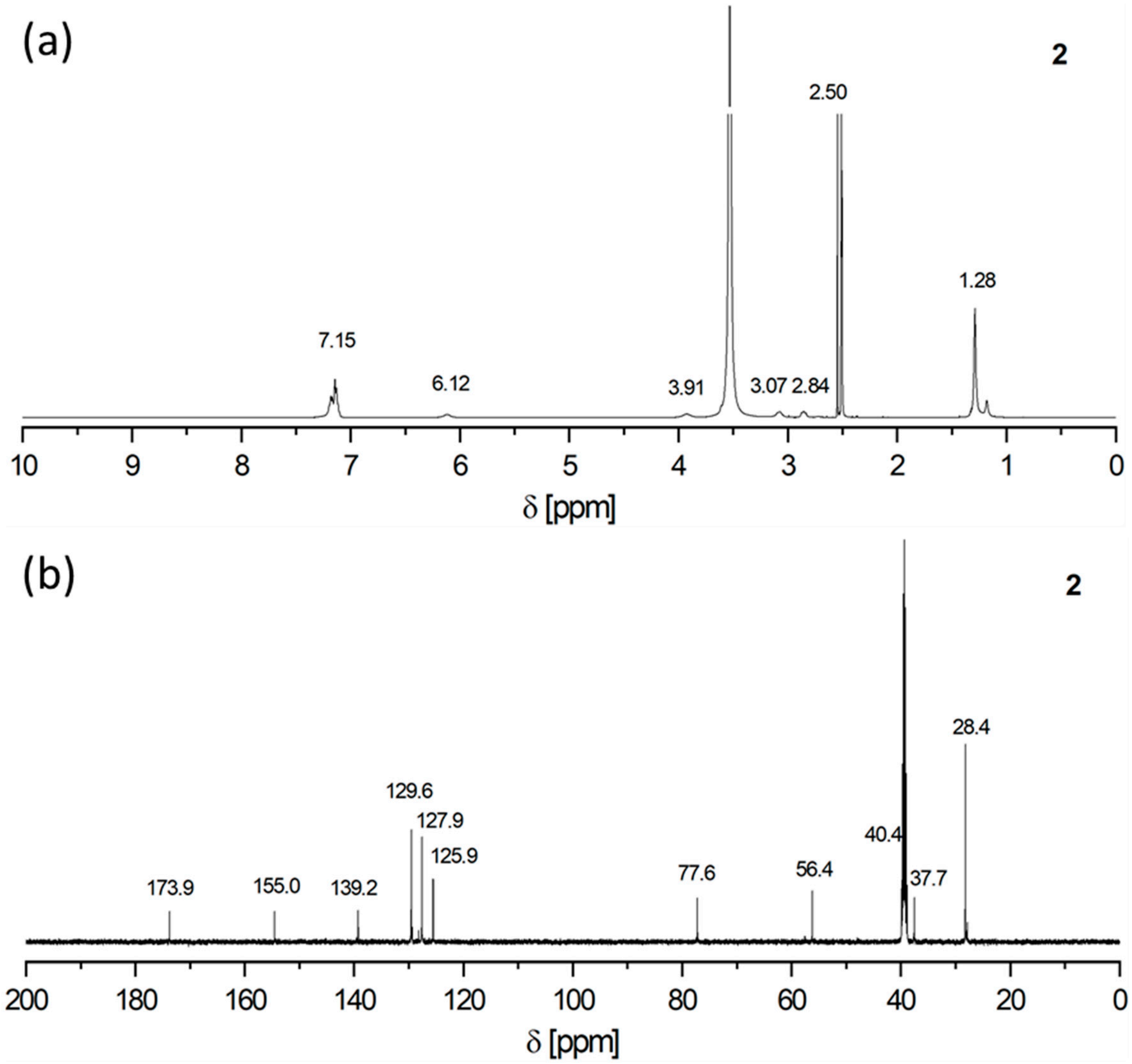

Figure S2. (**a**) ^{1}H NMR spectra (500.3 MHz, 298 K) of **2** in dmso-d$_6$. (b) ^{13}C NMR spectra (125.8 MHz, 298 K) of **2** in dmso-d$_6$.

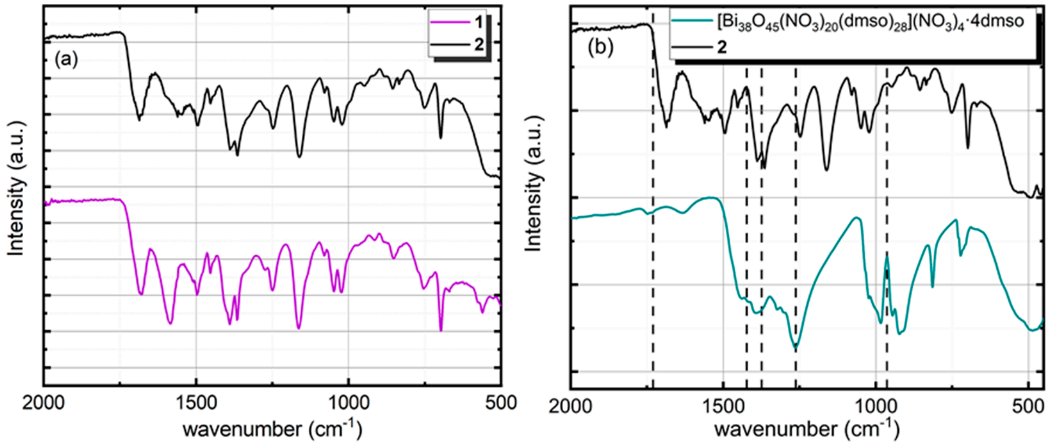

Figure S3. (**a**) FTIR spectra of Boc–Phe sodium salt (**1,** violet) and [$Bi_{38}O_{45}$(Boc–Phe–O)$_{24}$(dmso)$_9$] (**2**, black) powder, (**b**) FTIR spectra of [$Bi_{38}O_{45}(NO_3)_{20}$(dmso)$_{28}$]$(NO_3)_4$·4dmso (**A**, green) and [$Bi_{38}O_{45}$(Boc–Phe–O)$_{24}$(dmso)$_9$] (**2,** black) powder.

Table S1. FTIR vibration modes of $[Bi_{38}O_{45}(NO_3)_{20}(dmso)_{28}](NO_3)_4{\cdot}4dmso$ **A** associated with the FTIR spectrum in Figure S3(b).

Mode	Wavenumber / cm^{-1}
$\nu_{sym.\ mono}(NO_2)$	995
$\nu_{sym.\ bi}(NO_2)$	1265
$\nu_{as.\ bi}(NO_2)$	1380
$_{vas.\ mono}(NO_2)$	1420
$\nu(N{=}O)$	1735

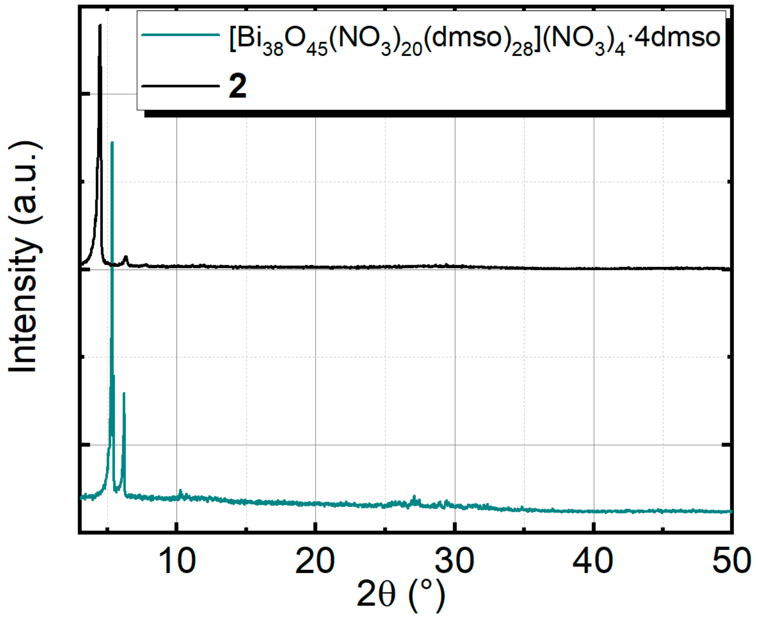

Figure S4. PXRD pattern of $[Bi_{38}O_{45}(NO_3)_{20}(dmso)_{28}](NO_3)_4{\cdot}4dmso$ (**A**, green) and $[Bi_{38}O_{45}(Boc–Phe–O)_{24}(dmso)_9]$ powder (**2**, black).

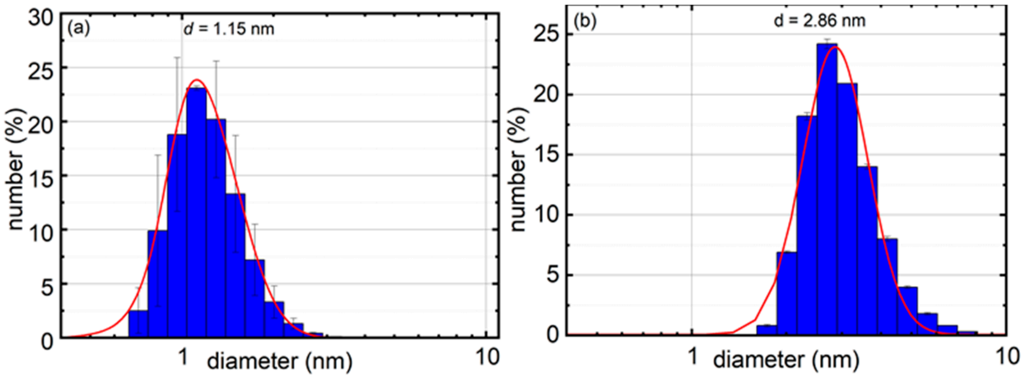

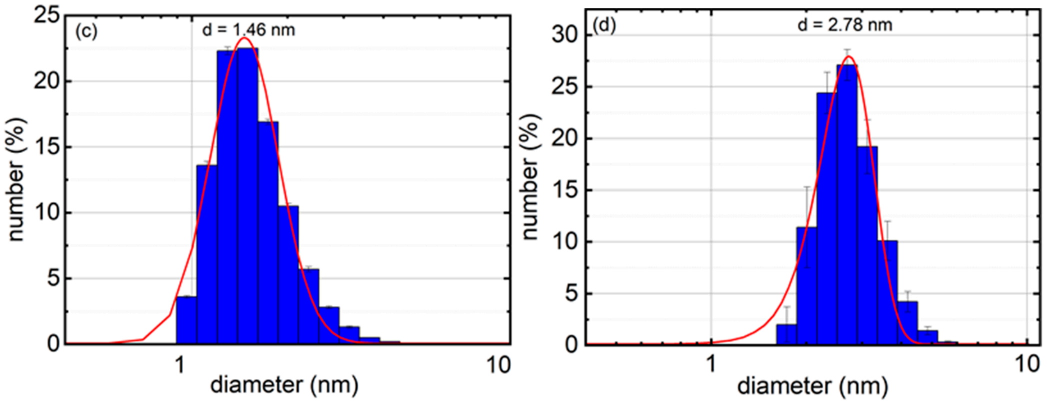

Figure S5. Exemplary particle size distribution (PSD) of $[Bi_{38}O_{45}(NO_3)_{20}(dmso)_{28}](NO_3)_4 \cdot 4dmso$ (**A**) (**a**) and **2** in dmso (**b**), in acetonitrile (**c**) and in ethanol (**d**). The data for PSD curve of **A** were used with permission from ref [63].

Table S2. FTIR vibration modes of **2** associated with FTIR spectrum in Figure 1 and Figure S6 (a) **2**, (b) **2**–DrA, (c) **2**–DrE, (d) **2**–SpA and (e) **2**–SpE (f) **2**–DrA–2 (g) **2**–DrE–2 (values given in cm^{-1}).

Mode	(a)	(b)	(c)	(d)	(e)	(f)	(g)
$\nu_{\text{Bi–O}}$	582	576	583	575	577	575	575
$\delta_{monosubst.}$ Aromat	699,753	699,752	701,754	699,752	700,752	699,753	702,753
$\nu_{\text{S=O}}$	948	946	945	947	947	947	947
$\delta_{\text{C–H}}$ (*C=C–H*)	1018	1021	1026	1021	1022	1022	1022
$\nu_{\text{C–N}}$ / $\nu_{\text{C–O}}$	1047,1162	1050,1165	1053,1170	1050,1162	1051,1164	1051,1165	1051,1165
$\nu_{\text{C–O–C}}$	1246	1246	1251	1247	1247	1247	1247
$\delta_{\text{C–H}}$(CH_3)	1363,386	1364,1387	1368,1393	1363,1389	1365,1389	1365,1387	1367,1387
$\nu_{\text{C=O}}$ carboxylate	1465,1554	1495,1553	1497,1559	1495,1554	1496,1556	1496,1553	1496,1557
$\nu_{\text{C=O}}$ amide	1688	1685	1694	1689	1690	1685	1688
$\nu_{as.\text{C–H}}$	2928,2975	2929,2976	2943,2978	2929,2977	2928,2977	2931,2978	2929,2978

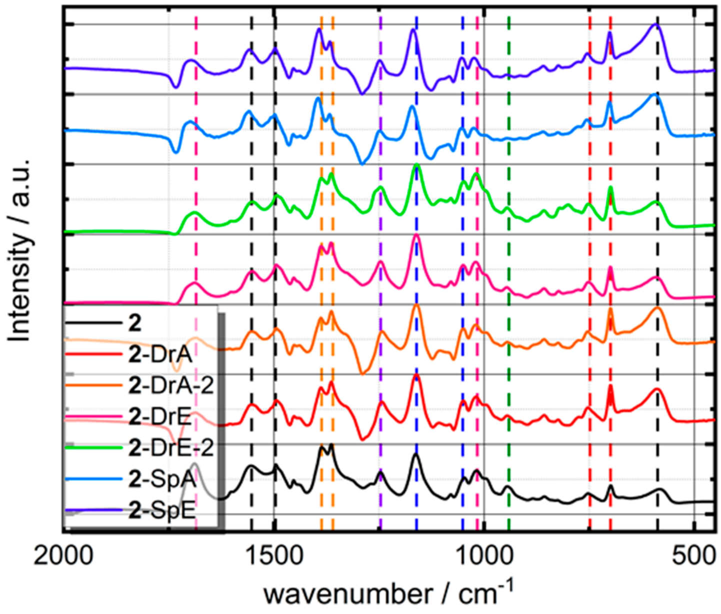

Figure S6. FTIR spectra for drop and spin coated films, **2** dissolved in ethanol (DrE and SpE) and acetonitrile (DrA and SpA) and double coated films from **2** dissolved in ethanol and acetonitrile for drop coated samples **(2**–DrE–2 **and 2**–DrA–2).

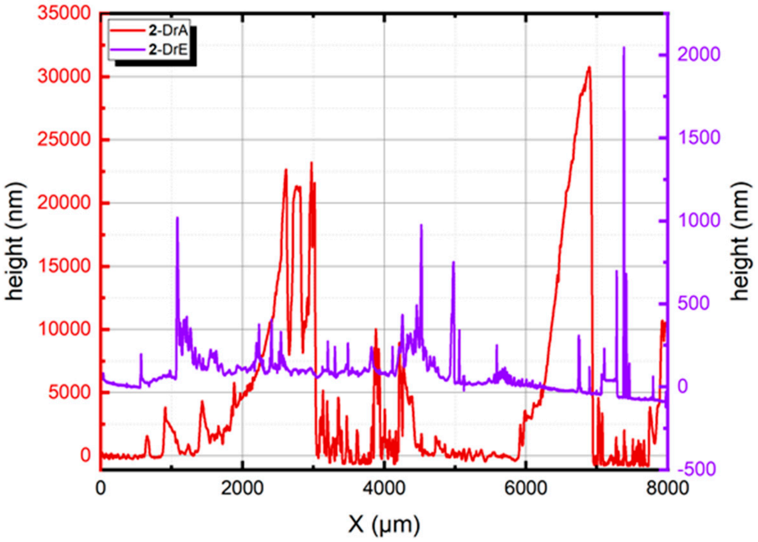

Figure S7. Profilometry of deposited films from bismuth oxido cluster **2** dissolved in ethanol (DrE) and in acetonitrile (DrA), respectively.

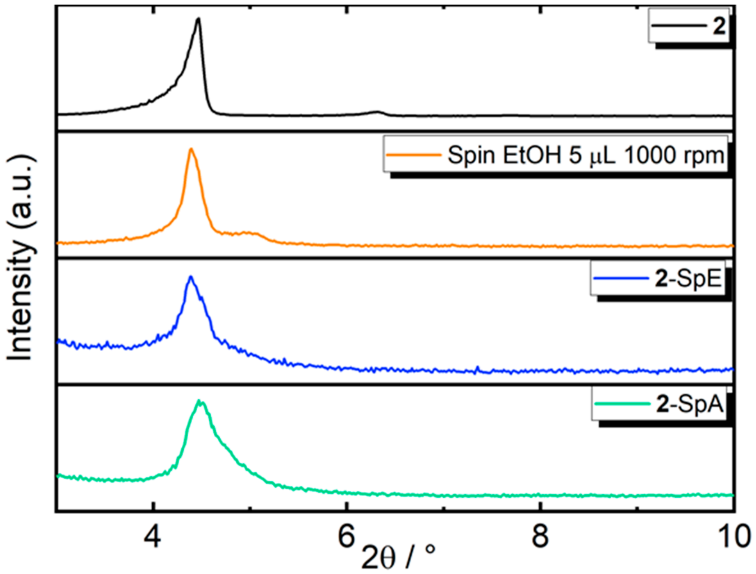

Figure S8. Comparison of XRD of compound **2** and drop coated films of **2** from ethanol (**2**–DrE) and acetonitrile (**2**–DrA) solution.

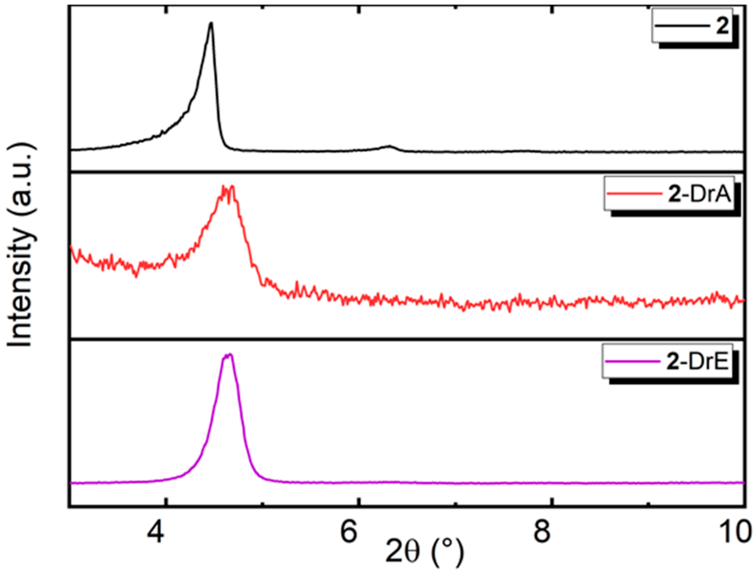

Figure S9. Comparison of XRD of compound **2**, spin coated samples of **2** from ethanol 5 μl and 1000 rpm, ethanol with 20 μl and 2000 rpm (**2**–SpE), and from acetonitrile 20 μl and 2000 rpm (**2**–SpA) solution.
